# Bioactive Properties and In Vitro Digestive Release of Cannabidiol (CBD) from Tailored Composites Based on Carbon Materials

**DOI:** 10.3390/pharmaceutics16091132

**Published:** 2024-08-27

**Authors:** Karol Zapata, Angie D. Vélez, Jorge A. Correa, Francisco Carrasco-Marín, Benjamín A. Rojano, Camilo A. Franco, Farid B. Cortés

**Affiliations:** 1 Bionatural Cosmeticos SAS, Medellín 050030, Colombia; bionaturalcosmeticos18@gmail.com; 2 Fenómenos de Superficie—Michael Polanyi, Facultad de Minas, Universidad Nacional de Colombia—Sede Medellín, Medellín 050036, Colombia; caafrancoar@unal.edu.co (C.A.F.); fbcortes@unal.edu.co (F.B.C.); 3 Química de los Productos Naturales y los Alimentos, Facultad de Ciencias, Universidad Nacional de Colombia—Sede Medellín, Medellín 050034, Colombia; advelezv@unal.edu.co (A.D.V.); brojano@unal.edu.co (B.A.R.); 4 Polyfunctional Carbon-Based Materials, UGR-Carbon, Department of Inorganic Chemistry, Faculty of Sciences, University of Granada, Avda. de Fuente Nueva, s/n, ES18071 Granada, Spain; fmarin@ugr.es

**Keywords:** bioavailability, bioactivity, carbon support, CBD, composites, digestive

## Abstract

The use of carriers to improve cannabidiol (CBD) bioavailability during digestion is at the forefront of research. The main objective of this research was to evaluate CBD bioactivity and develop CBD composites based on tailored carbon support to improve availability under digestive conditions. The antioxidant capacity of CBD was evaluated using spectrophotometric methods, and anti-proliferative assays were carried out using human colon carcinoma cells (SW480). Twenty-four composites of CBD + carbon supports were developed, and CBD desorption tests were carried out under simulated digestive conditions. The antioxidant capacity of CBD was comparable to and superior to Butylhydrox-ytoluene (BHT), a commercial antioxidant. CBD reflected an IC-50 of 10,000 mg/L against SW480 cancer cells. CBD in biological systems can increase the shelf life of lipid and protein foods by 7 and 470 days, respectively. Finally, acid carbons showed major CBD adsorption related to electrostatic interactions, but basic carbons showed better delivery properties related to electrostatic repulsion. A tailored composite was achieved with a CBD load of 27 mg/g with the capacity to deliver 1.1 mg, 21.8 mg, and 4 mg to the mouth, stomach, and duodenum during 18 h, respectively. This is a pioneering study since the carriers were intelligently developed to improve CBD release.

## 1. Introduction

Currently, approximately 60 different structures have been classified as cannabinoids, with tetrahydrocannabinol (THC) and cannabidiol (CBD) having higher amounts in plants. However, between these two substances, CBD has therapeutic properties, while THC can lead to psychoactive effects [1,2]. Nevertheless, the consumption of CBD as medicine is still a challenge because of its poor solubility in water, which reduces the options for oral consumption [3,4]. Today, the use of nanocarriers seeks to improve the dosing strategies used for active molecules such as CBD [5,6]. However, to date, the available reports are insufficient as they focus on engineering support materials to establish strong interacting complexes with high CBD loading but ignore the need to formulate reversible complexes that allow the controlled release of CBD. These reversible complexes are crucial to increasing the bioavailability of CBD from the supporting matrix when faced with multifarious physiological conditions. 

Wang et al. [7] reported the antioxidant activity of DPPH (2,2-diphenyl-1-picryl-hydrazyl-hydrate), ABTS (2,2′-Azino-bis (3-ethylbenzthiazoline-6-sulfonic acid), and FRAP (Ferric ion reducing antioxidant power) CBD-based nanocomplexes and described the release of CBD from the carrier. However, the in vitro release tests were carried out under simplified conditions using only digestive and duodenal enzymes; release tests in the mouth were ignored, as well as carrier engineering. A subsequent study by Wang et al. [8] established an experimental design for the formulation of an efficient CBD complex; however, release assays for all physiological conditions were again ignored. Years later, Wang et al. [9] evaluated the effect of lipid carriers during CBD immobilization and conducted CBD release trials under oral, gastric, and duodenal conditions. To date, this work is the closest to the present research; however, the supports proposed by the authors were of a lipid nature, which causes handling problems in the food industry, such as adherence to containers, lipid peroxidation of the carrier, and decreased oral distribution. In recent years, several studies have focused on complexes of a lipid nature [10,11,12,13,14]. Few authors have developed non-lipid supports. Söpper et al. [15] evaluated the development of silica-based nanocarriers for CBD loading. However, delivery trials throughout the entire gastrointestinal tract were ignored. Likewise, a single-carrier formulation was established, and no optimization test was executed. Fraguas-Sánchez et al. [16] developed CBD nanocomplexes based on poly-lactic-co-glycolic acid (PLGA) nanoparticles. Release studies were carried out in buffered saline solutions, and no organic or inorganic ingredient that simulated gastrointestinal conditions was used; therefore, it is impossible to determine the carrier’s stability under the relevant conditions. 

Many studies in the last four years have focused on developing non-lipid nanocomplexes ideal for CBD immobilization. However, to the best of our knowledge, no studies in the specialized literature report a carrier design that considers an appropriate relationship between adsorption on the support and desorption from the support under relevant physiological conditions [17,18,19,20]. In other words, most studies do not estimate the bioavailability (available vs. delivered) of the CBD molecule from the carrier; added to this, the release tests are incomplete when preparing buffered saline solutions or establishing a single condition (oral, stomach, or duodenal) such as digestion. In this study, the formulation of a support to promote CBD loading is as important as its formulation for the release of CBD from the composite. It is worth mentioning that the formulation should not be irreversible to guarantee the bioavailability and therapeutic effects of CBD under all physiological conditions. 

Carbonaceous-based materials have been widely used as adsorbents thanks to their large surface area, porous structure, adsorption properties, and chemical and thermal stability, as well as their regenerative, productive, and flexible characteristics. Carbonaceous materials have porous structures that provide large surface areas. This large surface area allows them to have more sites available to interact with the molecules they want to adsorb [21]. Furthermore, the porous structures of these materials allow for a high adsorption capacity. The pores act as traps for molecules, facilitating the capture of substances [22]. These materials have a high affinity for various compounds, including gasses, vapors, and liquids. This is due to van der Waals interactions and, in some cases, the ability to form hydrogen bonds with adsorbed molecules [23]. In addition, carbon-based materials are usually very stable both chemically and thermally. This means they do not easily break down or react with adsorbing substances, prolonging their life and effectiveness [24]. Many carbonaceous adsorbents can be regenerated and reused. This means that they can be treated to eliminate adsorbed substances and be used again, making them economic and ecological [25]. It is important to mention that they can be manufactured from various raw materials, and their properties are adjusted through different activation processes, such as steam or acid treatment. This allows adsorbents to be tailored for specific applications.

Hence, the objective of this research was to evaluate the biological properties of the CBD molecule (antioxidant, antiradial, and antioxidative) and develop CBD composites based on carbon materials of different physicochemical nature, determining the relationship between the CBD adsorption on the carbon support and the desorption from the composites at relevant physiological conditions. It is expected that the developed materials can open a wider landscape for easier use and formulation of CBD as a therapeutic solution for different health conditions.

## 2. Materials and Methods 

### 2.1. Materials

A standard solution of CBD (C_21_H_30_O_2_, 1.0 mg/mL in methanol, analytical standard, for drug analysis, MW: 314.46 g/mol), ethanol, methanol, Trolox, DPPH (2,2-diphenyl-1-picryl-hydrazyl-hydrate), ABTS (2,2′-azino-bis (3-ethylbenzthiazoline-6-sulfonic acid)), tetrazolium red, FeCl_3_, terephthalate (TH), fluorescein (FL), 2′,7′-Dichlorofluorescein diacetate (DCHF) probe, AAPH (2,2′-azobis(2-methylpropionamidine) dihydrochloride) probe, FeCl_2_, and Ethylenediaminetetraacetic acid (EDTA) (≥99% analytic grade) were provided by Sigma Aldrich (St. Louis, MO, USA) and employed for antioxidant and antiradical analysis. Linseed oil, collagen, and urea (≥80% purity) were purchased locally (Medellin, Colombia) and used in the biological oxidative assays. Resorcinol, formaldehyde, Cesium Carbonate, Barium Acetate, Phosphoric Acid, Ammonium Phosphate, Melamine, heptane, and Span 80 (≥99% analytic grade) were purchased from Sigma Aldrich (St. Louis, MO, USA) and used for the preparation of carbon supports. Finally, simulated physiological media were formulated using NaCl, NaH_2_PO_4_, KCl, CaCl_2_, NH_4_Cl, glucose, bovine serum albumin (BSA), pepsin, HCl, NaHCO_3_, KH_2_PO_4_, MgCl_2_, and NaOH (≥99% analytic grade) obtained from Sigma Aldrich (St. Louis, MO, USA). 

### 2.2. Methods 

#### 2.2.1. Cannabidiol (CBD) Identification 

The CBD absorption spectrum was measured from 200 to 700 nm using a Genesys 20 Thermo Scientific (Waltham, MA, USA) UV-Vis spectrophotometer to determine the maximum absorbance wavelength. A calibration curve was obtained at 255 nm for different concentrations of CBD in ethanol (100 and 3000 mg/L).

#### 2.2.2. Cannabidiol (CBD) Antioxidant Capacity

Total antioxidant activity was evaluated by in vitro colorimetric methods using DPPH, ABTS, and FRAP probes suggested by Brand-Williams et al. [26], Re et al. [27], and Benzie et al. [28], respectively. These probes change color because of the reception of radical hydrogen from antioxidant molecules. For the tests, 50 μL of diluted CBD and 950 μL of the probes in solution were mixed and incubated at room temperature in the dark for 30 min. The color change was measured at 517, 734, and 590 nm for DPPH, ABTS, and FRAP, respectively, using a Genesys 20 Thermo Scientific UV-Vis spectrophotometer (Massachusetts, United States). The results were compared with those of the reference antioxidant Trolox and expressed as μmol Trolox equivalents per 100 g CBD (μmol ET/100 g CBD).

#### 2.2.3. Cannabidiol (CBD) Antiradical Capacity

The antiradical activity was evaluated by in vitro fluorometric methods using TH, FL, and DCHF probes to determine the trapping of radicals’ hydroxyl OH^●^, Peroxyl ROO^●^, and total reactive oxygen species (ROS), as suggested by Yang and Guo [29], Ou et al. [30], and Martín-Romero et al. [31]. The OH^●^ radicals were generated through the Fenton Fe^2+^-EDTA-H_2_O_2_ reaction, while the radical initiator AAPH was employed to create Peroxyl ROO^●^ and ROS. When the radicals were complexed with the target probes, their fluorescence profiles were altered, and the ability of CBD to avoid alteration was monitored and compared with a control scavenger. Dimethyl sulfoxide molecules were used as OH^●^ scavengers, while Trolox was used as a reference for ROO^●^ and total ROS tests. For the assays, 50 μL of diluted CBD, 50 μL of radical-generating solution, and 2900 μL of solution containing the probe were used. The samples were incubated for at least 15 min in the dark, and fluorescence readings were obtained using a Perkin Elmer (Waltham, MA, USA) LS45 spectrofluorimeter. The results were compared with the reference scavenger and expressed as μmol scavenger equivalents SE per 100 g CBD (μmol SE/100 g CBD).

#### 2.2.4. Cannabidiol (CBD) Anti-Proliferative Capacity

To determine antiproliferative activity, the MTT (3-(4,5-dimethylthiazol-2-yl)-2,5-diphenyltetrazolium bromide) method was used, which is a colorimetric assay that allows the measurement of cellular metabolic activity. Under certain conditions, NADPH-dependent cellular enzymes can reflect the number of viable cells. These enzymes reduce tetrazolium dye to its insoluble purple formazan form. However, loss of cellular life is measured by the absence of the enzyme and the inability of the system to change its color. The method described by Bahuguna et al. [32] was used in this study. For this purpose, an isotonic saline solution was used to prepare CBD at 10, 100, 1000, and 10,000 mg/L, and the viability of human colon carcinoma cells (SW480) in the presence of CBD solutions was evaluated after 24 and 48 h of the assay.

#### 2.2.5. Cannabidiol (CBD) in Oxidative Stability 

The method proposed by Zapata et al. [33] was used to evaluate the ability of CBD to protect against oxidative deterioration in complex systems. Linseed oil and collagen were used as biological models to represent lipid and protein systems, respectively. To induce oxidation, the systems were subjected to accelerated degradation conditions by injecting air at a flow rate of 1150 mL/min at a temperature of 99.0 ± 0.1 °C and 10 mg/L of sulfuric acid for 60 min. A system without CBD was used as a negative control. To evaluate lipid oxidation, polar volatile compounds (PCs) were measured using a Testo 270 (Baden-Württemberg, Germany) submersible cooking oil tester. This device measures the dielectric constant of the oil, which is related to the amount of polar compounds generated during its oxidative deterioration [29]; the results are reported as % PC. To assay protein oxidation, the total carbonyls (TCs) were measured. To determine carbonyls, the spectrophotometric method described by Fagan et al. [34] was used with some modifications, and the oxidized extracts were mixed with dinitrophenyl hydrazine (DNFH) and incubated for 15 min in the dark. Finally, the increase in absorbance of the DNFH–carbonyl complex was measured at 370 nm. The results were expressed in nmol of carbonyls/mg of protein.

#### 2.2.6. Synthesis of Composites Based on Carbon Materials

##### Synthesis of Carbon Xerogels by Direct Emulsion 

The protocol of Bailón-García et al. [35] with some modifications was used for the synthesis of organic xerogels by direct emulsion (DE). The organic xerogels were prepared by the polycondensation of resorcinol with formaldehyde in an aqueous medium using the polymerization catalysts CsCO_3_ (Cs) and BaC_4_H_6_O_4_ (Ba). The resorcinol (24.8 g) and polymerization catalyst, 0.073 g of Cs, and 0.058 g Ba were dissolved in deionized water (1000 mL) using a three-neck glass reactor (2 L). The temperature was adjusted to 65 °C under stirring (480 rpm), and then 36.5 g of formaldehyde solution was dripped. The formed gels were aged at 65 °C for 24 h, filtered, and placed in acetone for 3 days to reduce porosity collapse during the subsequent microwave drying process. The gels were dried by microwave heating in an MS23J5133AG/AP oven (Suwon, South Korea) under an argon atmosphere for 1 min at 384 W until a constant weight was achieved. The pyrolysis of the organic xerogels was carried out at 900 °C for 2 h under N_2_ flow (300 cm^3^/min) using a Tube Furnace KJ-T1400 (Zhengzhou, China). The carbon xerogels obtained by direct emulsion catalysis with Cs and Ba were labeled as DECs and DEBa, respectively.

##### Synthesis of Carbon Xerogels by Inverse Emulsion 

For the synthesis of organic xerogels by inverse emulsion, the protocol of Zapata-Benabithe et al. [36], with some modifications, was used. The organic xerogels were prepared via polycondensation. Resorcinol (24.8 g) and the polymerization catalyst (0.073 g of Cs and 0.058 g of Ba) were dissolved in deionized water (33.8 mL) and 36.5 g of formaldehyde solution. The polycondensation reaction was performed at 65 °C for 62 min. The gels were then emulsified by pouring into heptane (1000 cm^3^) containing Span 80 as a surfactant using a three-neck glass reactor (2 L). The suspensions were stirred at a constant speed of 480 rpm for 20–24 h at 65 °C, filtered, and immersed in acetone for three days. Subsequently, the gels were dried using a microwave MS23J5133AG/AP oven (Suwon, South Korea) under argon atmosphere for 1 min at 384 W until a constant weight. Finally, the samples were carbonized using the procedure described above. The carbon xerogels obtained by inverse emulsion catalysis with Cs and Ba were labeled as IECs and IEBa, respectively.

##### Synthesis of Carbon Xerogels in Pellets 

For the synthesis of carbon xerogel pellets, the protocol of Morales-Torres et al. [37] with some modifications was used. The organic xerogels were prepared by polycondensation of resorcinol with formaldehyde in an aqueous medium. Resorcinol (24.8 g) and the polymerization catalyst, 0.073 g of Cs, and 0.058 g of Ba were dissolved in deionized water (1000 mL); the mixture was then placed in glass molds (30 cm length × 0.5 cm inner diameter) sealed and cured as follows: 24 h at 25 °C, 24 h at 50 °C, and, finally, 72 h at 80 °C. After the curing cycle, the xerogel rods were cut into 2 cm tablets. The drying and carbonization methods for organic xerogels in pellets were similar to those described above. The carbon xerogels obtained from pellets catalyzed with Cs and Ba were labeled as PLCs and PLBa, respectively.

#### 2.2.7. Surface Modification of Carbon Supports

All carbon supports were dried at 110 °C for one day before modification. The method used for modification was incipient impregnation, as described by Bailón-Garcia et al. [38] with some modifications. Surface modifiers were prepared as follows: an appropriate amount of modifier (Phosphoric Acid, Melamine, and Ammonium Phosphate) was dissolved in a minimum amount of water or ethanol (depending on the solubility) to add heteroatoms of phosphorus (P), nitrogen (N), and nitrogen–phosphorus (NP). The MA solutions were slowly dropped onto the support until completely moistened; the impregnated supports were dried under an infrared lamp for 24 h and subsequently fixed at 700 °C for 1 h with N_2_ as an inert atmosphere. The final concentration of MA in the support was 4% of this dry weight. 

Finally, 24 materials were obtained using two catalysts, CsCO_3_ (Cs) and BaC_4_H_6_O_4_ (Ba), three synthesis methods, direct emulsion (DE), inverse emulsion (IE), and pellet formation (PL), and four surface modifiers as controls (without modifiers), phosphorus (P), nitrogen (N), and nitrogen–phosphorus (NP).

#### 2.2.8. Characterization of Carbon Supports

To determine the physical and chemical properties of the carbon supports, their shapes, surface areas (S_BET_), and superficial charge densities were determined using scanning electron microscopy (SEM), N_2_ adsorption isotherms (Iso-N_2_), and point zero of charge (PZC), respectively. An LEO GEMINI-1530 Carl Zeiss microscope (Berlin, Germany) was used for the SEM analysis. At the same time, for PZC determination, each carbon was dispersed in pH between 1 and 14 using NaOH (0.1 M) and HCl (0.01 M) stock solutions; each dispersion was taken to the NanoPlus 3 equipment (Norcross, ATL), and its Zeta Potential was determined. The pH vs. Zeta Potential relationship was plotted on the abscissa and ordinate, respectively, and the intersection point with the abscissa was considered the point zero of charge (PZC), that is, the pH at which the particle presents a zero-charge density. Finally, the surface area (S_BET_) was calculated from N_2_ sorption isotherms for carbons using a Quadrasorb SI instrument (Boynton Beach, FL, USA) and applying the Brunauer–Emmett–Teller Equation [39] to the isotherms. 

#### 2.2.9. Adsorption Isotherms

The composites were formed following the protocol reported by Zapata et al. [40] with some modifications, as follows: 100 mg of carbon supports and 10 mL of CBD ethanolic solution (600 mg/L) were mixed at 150 rpm and 25 °C for 10 h. Then, the impregnated carbons were centrifuged at 2000 rpm for 10 min, and the supernatant absorbance was measured at 255 nm to calculate the final CBD concentration; subsequently, the concentration of CBD on carbon supports was calculated using Equation (1), and the results were expressed as mg CBD/g carbon support.

(1) $$CBD\;on\;carbon\;supports=\frac{\left[Ci-Cf\right]\;\times \;V}{M}$$

where Ci is the initial concentration of CBD, Cf is the final concentration of CBD, V is the assay volume, and M is the mass of carbon support. 

To evaluate the phenomenology of the carbon+CBD interaction, adsorption isotherms were constructed using Batch-type setups at 25 °C, varying the concentration of CBD in 10 mL of ethanol and keeping the amount of carbon constant (100 mg). The suspensions were shaken at 200 rpm and allowed to equilibrate for 24 h. Then, the amount of CBD adsorbed on the carbon surface was determined by UV-Vis spectrophotometry, the calibration curve at 255 nm previously constructed (Absorbance = 0.9826 × CBD Concentration in g/L − 0.1131), and Equation (1). The adsorption isotherm was constructed through the relationship between the adsorbed quantity of CBD (Nads) and the un-adsorbed amount (Ce) in each assembly, according to the method described by Franco et al. [41], and the experimental data were fitted to the SLE model [42].

#### 2.2.10. Complete Factorial Design

To determine the factors that most influence the immobilization of CBD on carbon supports, a complete 2 × 3 × 4 factorial design was used. The selected factors were the type of catalyst with two levels (Cs and Ba), type of synthesis with three levels (direct, inverse, and monolith synthesis), and a surface modifier with four levels (without modification, modified with nitrogen, modified with phosphorus, and modified with nitrogen–phosphorus). The results of the factorial design were analyzed using the statistical software R Free Version (Vienna, Austria), showing the effect of the factors on the adsorbed amount of CBD on the carbon support (mg/g) through an analysis of variance (ANOVA) with a significance level of α = 0.01 and an F means test corrected by Holm’s method [43] using the simple function and interaction graphs.

#### 2.2.11. Release Assays under Simulated Physiological Conditions

To simulate oral conditions, 1 mL of human saliva was mixed with 5 mL of water and maintained at 37 °C under constant stirring at 55 rpm until use. Stomach conditions were simulated with a 2:1 mixture of inorganic and organic solutions. The inorganic solution was composed of 2.2 g of NaCl, 1.1 g of NaH_2_PO4, 1.1 g of KCl, 0.3 g of CaCl_2_, and 0.4 g of NH_4_Cl, while the organic solution was composed of 0.43 g glucose, 0.17 g urea, 0.2 g bovine serum albumin (BSA). and 0.5 g of pepsin equivalent per liter of water. The pH was adjusted to 2.1 with HCl 0.1 M, and the mixture was homogenized at 55 rpm until use. Finally, to simulate duodenal conditions, an inorganic solution composed of 3.7 g of NaCl, 1.8 g of NaHCO_3_, 0.2 g of KH_2_PO_4_, 1.9 g of KCl, and 0.1 g of MgCl_2_ per liter of water was prepared. Then, 0.02 g urea, 0.2 g CaCl_2_, and 0.2 g albumin were added, and the mixture was homogenized by stirring at 55 rpm. The pH of the solution was adjusted to 7.4 with NaOH 0.1 M until use. For the release tests, the carbon support with the highest CBD adsorption during composite formation was used. To carry out the delivery experiments, 100 mg of the optimal composites was exposed to 10 mL of oral medium for 5 min, 10 mL of stomach solution for 5 h, and 10 mL of duodenal solution for 18 h. The assays were conducted at 110 rpm and 37 °C to simulate the movement and gastrointestinal transit of food from the mouth to the intestine. At each stage, the simulated medium was analyzed to determine the amount of CBD released using spectrophotometry at 255 nm, as previously described.

#### 2.2.12. Statistical Analysis

All assays were performed in triplicates. The reported results are expressed as the mean ± standard deviation of the measurements. To evaluate the effect of the independent variables, an ANOVA was performed with a p level of significance equal to 0.01. The software used for the analysis was the free version of Statgraphics Centurion 18 (Statgraphics Technologies, Inc.). For results with statistically significant differences, alphabetical superscripts were used. Likewise, error bars were added to the data for each curve.

## 3. Results and Discussion 

### 3.1. Cannabidiol (CBD) Bioactivity

#### 3.1.1. Antioxidant and Anti-Radical Capacity

Antioxidant capacity is the ability of a compound to reduce a certain number of free radicals to avoid their damaging effects on living tissues. The antioxidant capacity of CBD was determined using the FRAP reducing power and the capture of ABTS and DPPH radicals. Table 1 summarizes the obtained results. The ABTS values were higher than the DPPH and FRAP values, which is associated with difficulty accessing the active centers of DPPH and FRAP, unlike ABTS. Therefore, it is difficult to achieve the orientation of CBD and DPPH or FRAP molecules for the transfer of electrons between them. In a similar study carried out on different THC and CBD molecules, a greater antioxidant capacity measured by the ABTS assay was also observed [44].

The antioxidant activity of CBD can be attributed to the phenolic group present in its structure, which has two -OH groups that can easily donate hydrogen atoms or electrons (inductive effect) to stabilize radicals, added to its cyclic structure that allows the resonance of unpaired electrons after the formation of the phenoxyl radical, interrupting the propagation of radicals (resonant effect) [45]. To place the CBD antioxidant results in context, they were compared with those reported for butylated hydroxytoluene (BHT), a widely used commercial antioxidant. The ABTS assay for CBD indicated that it had a similar capacity to that of BHT [46]. However, the DPPH and FRAP results were lower than those for BHT [47,48], which is related to CBD’s more voluminous structure compared with that of BHT and its steric complexity to attack voluminous radicals. However, in biological tissues, the presence of small and bulky antioxidants is necessary to attack all types of radicals, from small reactive oxygen species generated during cellular respiration [49] to radicals such as oxidized biomolecules [50]. Spectrophotometric tests are the first approach for determining the antioxidant quality of samples. However, the evaluation of ROS trapping as initiators of biomolecule oxidation is a relevant physiological approach to the performance of CBD. Table 2 presents the ability of CBD to trap total ROS, hydroxyl radicals ^●^OH, and peroxyl radicals ROO^●^.

Table 2 shows the antiradical activity results using BHT as a reference. CBD presented an ORAC value 2.4 times higher than that of BHT. Regarding the trapping capacity of total ROS, CBD is comparable to BHT, whereas the scavenging capacity of ^●^OH radicals is six times better for CBD. These results are promising because BHT is a synthetic antioxidant commonly used in food, cosmetics, and pharmaceutical products to prevent oxidation and extend the shelf life of these products. However, recent reports [51] have indicated that BHT can have significant toxicological effects. 

Animal studies have shown that high doses of BHT can induce liver damage, including hepatomegaly and alterations in liver enzyme levels. Other studies [52] have suggested that BHT may be genotoxic and promote tumor development in certain contexts. Evidence suggests that BHT can interfere with thyroid function and reproduction. Animal studies have indicated hormonal alterations and adverse effects on fertility [53]. It has also been suggested that BHT may have neurotoxic effects [54]. Finally, there are reports [55] that exposure to BHT can cause respiratory irritation and allergic skin reactions in humans. 

Despite these potential adverse effects, BHT continues to be approved by several regulatory agencies as a safe additive within the established consumption limits. However, continuous evaluation and caution regarding its use are recommended. Therefore, the discovery of natural CBD-type molecules with superior antioxidant performance and no toxicological effects has become an alternative to synthetic antioxidants. Finally, several studies have demonstrated the efficacy of CBD as a therapeutic molecule [56,57,58]. For example, given its ability to be selective cytotoxic, CBD seems to be focused on inhibiting the characteristics of cancer cells, including rapid DNA replication, altered metabolism (Warburg effect), elevated expression of receptors or markers on the surface, and acidic and hypoxic microenvironment, among others. 

#### 3.1.2. Anti-Proliferative Capacity

**Figure 1:**
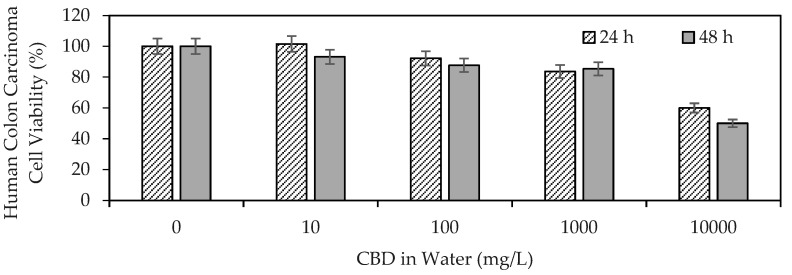
Viability of human colon carcinoma cells (SW480) exposed to CBD in solution at concentrations between 10 and 10,000 mg/L evaluated by the MTT (3-(4,5-dimethylthiazol-2-yl)-2,5-diphenyltetrazolium bromide) method.

Figure 1 shows the results of CBD anti-proliferative activity on human colon carcinoma cells (SW480).

The results demonstrated the ability of CBD to influence the viability of human colon carcinoma cells, highlighting its antiproliferative properties. CBD reflected an IC50 of >10,000 mg/L (>32 μM) at 24 h, whereas the mean lethal dose using CBD at 48 h was ~10,000 mg/L (32 μM). The IC50 (half inhibitory concentration) is the concentration of CBD necessary to inhibit the biochemical function of SW480 cancer cells by 50%. Medications such as Doxorubicin (adriamycin), imatinib (Gleevec), gefitinib (Iressa), trastuzumab (herceptin), and cisplatin, used today in anticancer therapy, report IC50 values of up to 10 μM [59]. Although the IC50 values were on the same scale, they were below those found in the present study for CBD. These findings provide a starting point for the development of cannabinoid-based anticancer drugs. The antiproliferative effect of CBD has been associated with its ability to promote cancer apoptosis [60], inhibit proliferation [61], reduce the invasion and metastasis of cancer cells [62], alter the tumor microenvironment [63], and induce autophagy [60]. 

#### 3.1.3. Oxidative Stabilization

**Figure 2:**
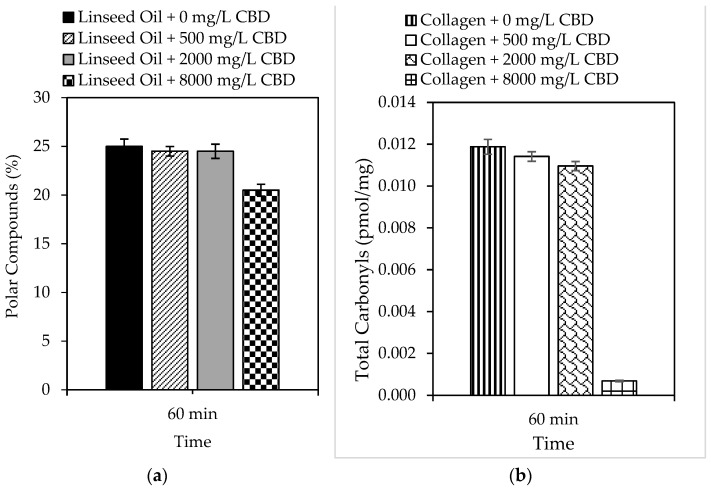
Formation of (**a**) polar compounds and (**b**) total carbonyls by accelerated oxidation of linseed oil and collagen in the presence of CBD (0 mg/L–8000 mg/L) at 99.0 ± 0.1 °C and 10 mg/L of sulfuric acid for 60 min with 1150 mL/min of air.

In the present study, the ability of CBD to prevent oxidation of complex systems such as lipids and proteins was also evaluated, and the results are shown in Figure 2a and 2b, respectively. Linseed oil was selected as a lipid model because of its high content of unsaturated fatty acids (>80%), which are susceptible to peroxidation [64]. Likewise, collagen was selected as a protein model because it plays multiple roles in meat muscle. Similarly, both models can predict CBD behavior under physiological conditions. The results demonstrated the ability of CBD to inhibit the peroxidation of linseed oil, which was subject to the concentration of CBD in the model system. For example, reductions of up to 18% in 60 min were reported using 8000 mg/L CBD. Likewise, the ability of CBD to inhibit protein oxidation was also dependent on concentration; reductions of up to 94% were reported using 8000 mg/L CBD.

A general approach was used to determine the number of days the shelf life of a specific food increases using an antioxidant that reduces lipid peroxidation by 18%. Assuming a constant peroxidation rate, we can say that in 30 days, peroxidation reached 100% of the critical level. The peroxidation rate was 3.33% per day. An antioxidant that protects against lipid peroxidation by 18%, such as CBD, reduces the daily rate of peroxidation by 18%. This means that the new daily rate would be 82% of the original, that is, 2.7% per day. With the new peroxidation rate, the number of days it took to reach 100% of the critical level was calculated, and it was concluded that CBD in polyunsaturated lipid systems (susceptible to oxidation) could increase shelf life from 30 to 37 days. If the same exercise was performed for protein systems, in which the incorporation of 8000 mg/L of CBD reduced oxidation by 94%, it can be concluded that the shelf life of a protein-based food could be increased from 30 days to 500 days. This is a simplified calculation, and the actual shelf life may vary based on other food-specific factors and storage conditions [65,66]. However, it provides a snapshot of CBD protection in complex biological systems. The biological stability results allow us to conclude that the inclusion of CBD in foods prevents rancidity caused by the oxidation of fats and oils, as well as the loss of nutritional qualities, by preventing the oxidation of fat-soluble vitamins contained therein. Finally, CBD, as a food antioxidant, can preserve the color of foods by preventing the oxidation of natural pigments, among others [67,68]. 

However, antioxidants in food can also strengthen the endogenous antioxidant system of consumers. For example, an antioxidant capable of inhibiting lipid peroxidation, such as CBD, can also prevent the oxidation of low-density lipoproteins (LDLs), which are key players in the formation of atherosclerotic plaques in arteries [69] and damage to blood vessel cells during the development of hypertension [70]. CBD also prevents the generation of mutagenic products from peroxidation, which damages DNA and contributes to cancer progression [71]. CBD inhibits the accumulation of lipid peroxidation products that can induce neuronal damage associated with Alzheimer’s disease [72]. Finally, the oxidative stress involved in the degeneration of dopaminergic neurons in Parkinson’s can also be reduced by CBD [73]. Similarly, antioxidants capable of protecting against protein oxidation have been associated with disease prevention. For example, CBD can prevent the oxidation of proteins such as beta-amyloid and tau proteins related to Alzheimer’s disease [74] and the oxidation of the alpha-synuclein protein that forms Lewy bodies, which are a hallmark of Parkinson’s disease [75]. CBD can inhibit the oxidation of superoxide dismutase and prevent the degeneration of motor neurons [76]. Type 2 diabetes mellitus, metabolic syndrome, nonalcoholic steatohepatitis, hepatitis, systemic lupus erythematosus, and rheumatoid arthritis are diseases associated with protein oxidation [77].

### 3.2. Composites Based on Carbon Materials

#### 3.2.1. Carbon Support Properties 

**Figure 3:**
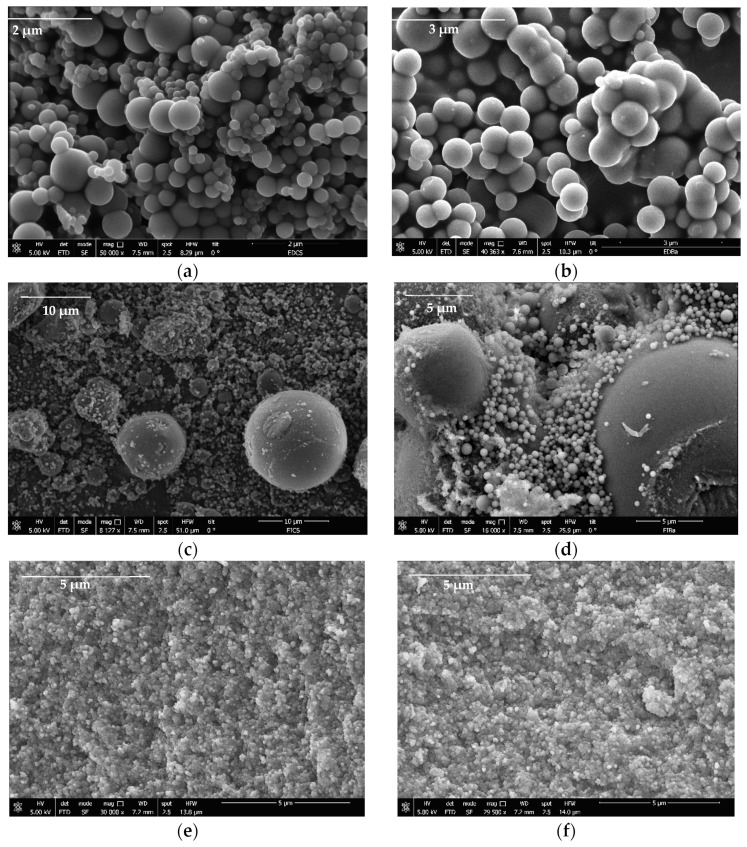
SEM images of (**a**) DECs, (**b**) DEBa, (**c**) IECs, (**d**) IEBa, (**e**) PLCs, and (**f**) PLBa carbon-based supports for the CBD immobilization.

In the present investigation, 24 different carbons were synthesized for use as supports to improve CBD biodistribution under physiological conditions. Characterization tests of the carbon supports were carried out to correlate their physicochemical properties with CBD immobilization capacity. Figure 3 shows the morphology of the base carbons (without surface modifications) obtained using scanning electron microscopy (SEM).

The results suggest that the carbons formed three-dimensional networks of agglomerated primary particles that have variable sizes from the micrometric regimen; the primary particles of the pellets were smaller (<1 μm) than those of the powder gels obtained by direct and reverse emulsion (>1 μm). All materials presented interparticle spaces with pore size on a micrometric scale where CBD molecules can be housed. On the other hand, to identify the fundamental physicochemical parameters in the CBD + carbon interaction, properties such as surface area (S_BET_) and point zero of charge (PZC) were assessed for all the materials. The results are presented in Table 3.

The porous area was affected by the functionalization of the material, which was expected because the chemical groups tend to lodge in free spaces, providing new chemical characteristics to the material. The unmodified and nitrogen-modified (N) carbons revealed basic surfaces with PZC greater than 7.0, which was due to the presence of only carbon structures and the inclusion of nitrogenous^+^ compounds that behave like bases given their ability to accept electrons, as stated in the Lewis acid–base theory. In contrast, carbons modified with phosphorus (P), or phosphorus–nitrogen (NP), presented PZC lower than 7.0, indicating acidic surfaces that are related to the presence of phosphate groups (PO_4_^3−^) rich in electrons to donate, which gives them surface acidity according to Lewis theory [78]. It is expected that materials with higher S_BET_ host a greater amount of adsorbate; however, this would be a simplistic conclusion if we consider that there are chemical mechanisms that are also involved in the interaction of the adsorptive couple. 

#### 3.2.2. CBD Adsorption on Carbon Supports 

**Figure 4:**
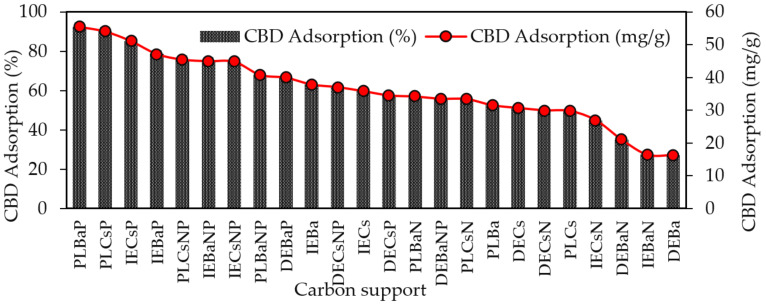
Adsorption capacity of CBD on carbon supports using 100 mg of carbon supports and 10 mL of CBD ethanolic solution (600 mg/L) at 150 rpm and 25 °C temperature for 10 h.

The results of the adsorption capacity of CBD on each of the carbon supports are shown in Figure 4. The highest CBD adsorption capacities were obtained for acidic materials (PZC < 7.0) modified with P or NP, with adsorption values of up to 92.5%, whereas the lowest adsorption values were reported for basic carbons, with a maximum adsorption of 63%. 

**Figure 5:**
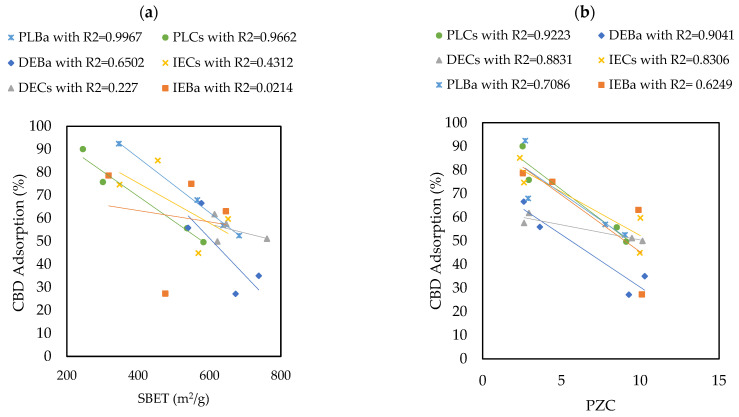
Pearson’s correlations showing the influence of (**a**) S_BET_ and (**b**) PZC properties on the ability of carbon supports to adsorb CBD.

The materials that adsorbed the most and the least CBD were PLBaP and DEBa, with adsorptions of 92.5% (55.5 mg/g) and 27.19% (16.3 mg/g) and with areas of 346 and 673, respectively, which allows us to conclude that adsorption does not seem to be determined by the area but by the chemistry surface of the carbon supports. To determine the influence of PZC and S_BET_ on the adsorptive capacity of the materials, Pearson’s correlations were calculated, and the results are shown in Figure 5.

**Figure 6:**
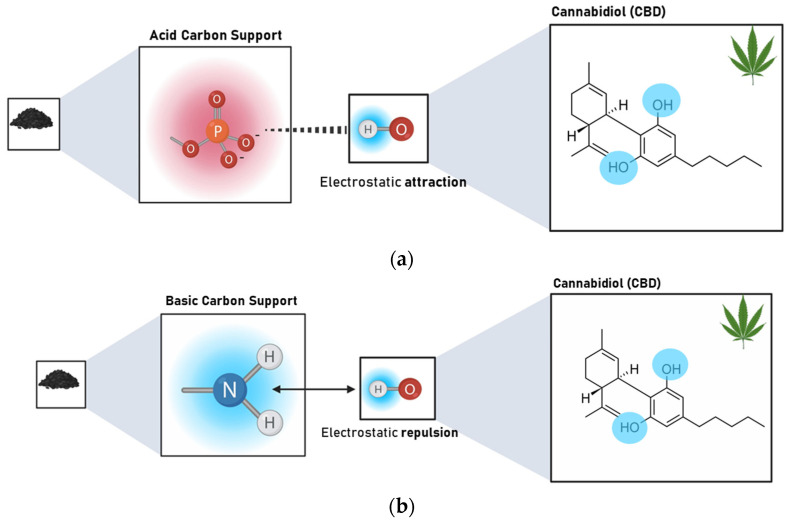
Representation of the interaction type between (**a**) acidic carbon supports (PZC < 3) modified with P and NP and CBD and (**b**) basic carbon supports (PZC > 7) unmodified and modified with N and CBD at the pH of the composite formation (pH = 6.5).

The results revealed that the Point of Zero Charge (PZC) has a greater influence on the adsorption capacity of CBD on carbon supports than S_BET_. The Pearson’s correlation coefficients between S_BET_ and CBD adsorptions were between 0.02 and 0.99, with an average of 0.548. In contrast, for the relationship between PZC and CBD adsorptions, these coefficients ranged between 0.62 and 0.92, with an average of 0.81. However, for pellet carbons, both S_BET_ and PZC appeared to be decisive in defining the adsorption of CBD. Similarly, the results indicated an inverse relationship between the PZC of the carbon supports and their CBD adsorption capacities. That is, acidic materials with PZC values lower than 7.0 (modified with P or NP) showed better adsorbent capacities, up to four times higher than those reported for other materials (Figure 4). To explain this phenomenon, it is necessary to consider that the carbon supports modified with phosphorus (P) and nitrogen–phosphorus (NP) presented an acid PZC < 3. This implies that at the pH of the CBD solution (pH = 6.5), the carbon support acquires a negative charge. This charge allows electrostatic interaction with CBD, which has a pKa of 9.13, and is positively charged; in this case, the intermolecular forces between the adsorbent pair would be of the ion–ion attraction (CBD^+^ vs. CARBON^−^). However, the use of unmodified carbon or carbon modified with nitrogen (PZC > 7) suggests an electrostatic repulsion between the adsorptive pair. A representation of this phenomenon is presented in Figure 6.

#### 3.2.3. CBD Release Assays from Carbon Supports 

**Figure 7:**
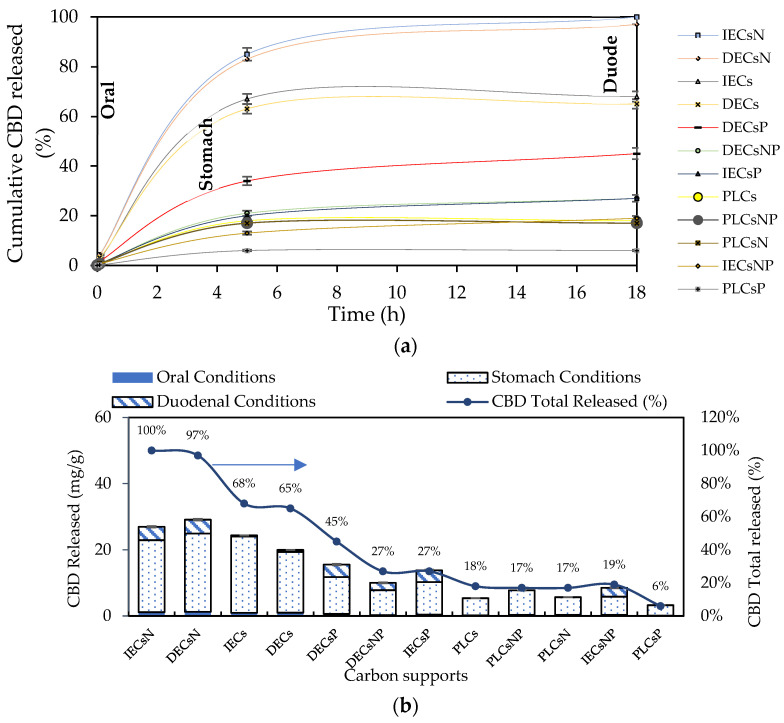
(**a**) CBD release kinetics from the CBD + carbon complexes to simulated oral, gastric, and duodenal physiological conditions at 37 °C and (**b**) CBD released from the complexes for each simulated oral, gastric, and duodenal physiological condition expressed as mg of CBD released per g of carbon support and % CBD released.

Twelve complexes were chosen for the release assays under simulated physiological conditions. In addition to the carbon support with the highest percentage of CBD adsorption (acids), others with intermediate and low adsorption (basic and neutral) were selected. The results of CBD release for the selected systems are shown in Figure 7. 

**Figure 8:**
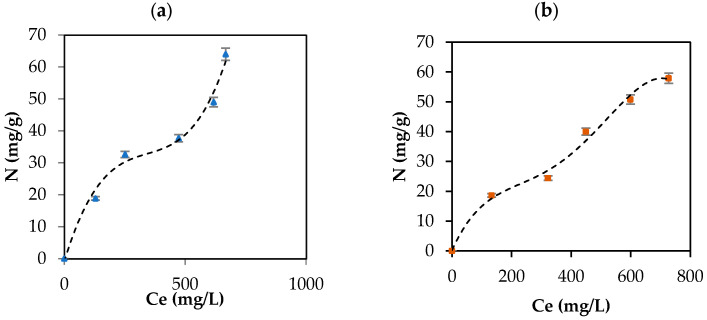
CBD adsorption isotherm on (**a**) IECsN (▲) and (**b**) DECsN (■) using 100 mg of carbon support and different concentrations of CBD at 25 °C and fit to the experimental data to SLE model (---).

CBD release from the carbon support occurred in the following order: IECsN > DECsN > IECs > DECs > DECsP > DECsNP > IECsP > PLCsNP > IECsNP > PLCsP. Interestingly, the results revealed that CBD was released in greater proportion from materials that presented lower adsorbent tendencies (Figure 4). These materials correspond to basic carbons generated in the absence of modifiers and the presence of nitrogen modifiers. In contrast, acidic materials presented difficulties in releasing CBD molecules under all physiological conditions evaluated. This is associated with the establishment of strong ion–ion attractions when using this type of adsorptive couple. In practical terms, while a material such as PLCsP has the capacity to adsorb 90.07% of the available CBD (54 mg/g), under simulated physiological conditions, it is only capable of releasing 6% of CBD (3.1 mg/g). Therefore, the bioavailability of the CBD active molecule when using the complex is less than 10%. Inversely, a material such as IECsN that adsorbed 45% of the available CBD by not establishing irreversible interactions could deliver 27 mg/g of CBD under physiological conditions, resulting in a bioavailability of 100%. According to the latest recommendations from the Food Standards Agency, adults should limit their consumption of CBD in food products to 10 mg per day [79]; therefore, the use of 370 mg of IECsN would provide the recommended dose. To achieve the same dose through the use of complexes based on PLCsP and analogs, between 1 and 3 g of product are required, which translates into greater costs and biological implications. Another significant point is that the composites based on IECsN and DECsN presented complete desorption through the three simulated physiological systems because of the electrostatic weakening of the adsorptive couple subjected to different conditions of pH, agitation, and ionic strength [7]. The kinetics of CBD release from the optimal materials, IECsN and DECsN, were fitted to the Higuchi model [80], and the adjustment coefficients R in both cases were greater than 0.90. This fitting allows us to conclude that CBD diffusion occurs in a single dimension and that the dissolution and swelling of the carbon are negligible. However, the diffusivity of CBD is constant, and perfect immersion conditions are always reached in the dissolution medium. The Higuchi model is widely used to describe the release of poorly soluble drugs, such as CBD, in aqueous media from a solid matrix as a carbon support. The model also allowed us to calculate the Higuchi dissolution constant (KH); the KH value was 0.044 and statistically the same for both materials, which translates into a CBD release of 0.044 mg per minute from 1 g of support. Finally, to understand the phenomenology of the interaction between IECsN and the DECsN carbon support and CBD molecule during the formation of the best composites, adsorption isotherms were obtained, and the results are shown in Figure 8.

According to UIPAC reports, the adsorption isotherms are type II, characteristic of macroporous solids such as carbon supports, which represents the adsorption of CBD in monolayers and multilayer without restrictions on the heterogeneous surfaces of the IECsN and DECsN carbon supports. The isotherms were concave at low relative concentrations and convex at high concentrations. Concentrations of 33 mg/L and 24 mg/L indicate the values for which the monolayer coating of the IECsN and DECsN carbon supports occurs, respectively, indicating the principle of multilayer adsorption. Based on the isotherms, the amount of CBD that can be adsorbed (N_max_) on carbon supports can be up to 100 mg/g, according to the SLE model [42]. For this type of material, an important conclusion is that type II isotherms occur for completely reversible adsorption, which would explain the bioavailability of CBD when it is immobilized on IECsN and DECsN supports.

## 4. Conclusions

In this study, the biological properties of CBD, such as its antioxidant capacity, ability to capture radical species, and antiproliferative effects, were successfully investigated. The results highlighted CBD as a phytochemical with comparable benefits and, in some cases, superior to those reported for widely used commercially used antioxidants such as BHT. After the biological properties of CBD were analyzed, 24 types of carbon supports with different physical and chemical characteristics were synthesized. The adsorption tests indicated that, in this case, the chemical properties of the carbon supports have a greater impact on the adsorption of CBD than their textural properties. Inverse correlations between point zero of charge and CBD adsorption were found in 90% of the cases (acidic carbon absorbed more CBD). However, as in most studies, an exclusive evaluation of the conditions that favor the formation of CBD + carbon composites is insufficient. The irreversibility of the composites and the conditions under which the active molecule CBD is released toward the target organs for which they were designed are unknown. The results of this study revealed that the ideal carbon supports for composite formulations showed lower CBD release rates under simulated physiological conditions (irreversible adsorption). Therefore, increasing the bioavailability of CBD is not recommended. In contrast, basic materials with intermediate adsorption released a greater amount of CBD during the digestive processes, which was facilitated by the intrinsic electrostatic repulsion between the adsorbent couple. These pioneering findings suggest that we should be careful when formulating CBD-based complexes to increase their solubility and availability. 

## Figures and Tables

**Table 1:** Antioxidant capacity of CBD measured by in vitro spectrophotometric techniques, DPPH, ABTS, and FRAP at 25 °C. All assays were performed in triplicate n = 3; in all cases, values are statistically different.

Technique	TEAC (μmol Trolox/100 g CBD)
ABTS	79,609 ± 2586
DPPH	3385 ± 63
FRAP	5259 ± 194

**Table 2:** The antiradical capacity of CBD measured by in vitro fluorimetric techniques, including the DCFH assay, terephthalic assay, and ORAC assay, at 37 °C and phosphate buffer solution (PBS) at pH = 7.4. All assays were performed in triplicate n = 3; in all cases, the values are statistically different.

Technique	Radical	Result (per 100 g)	
		CBD Sample	BHT Reference
DCFH assay	Total ROS	4236 ± 213 μmol equivalents Trolox	5143 ± 239 μmol equivalents Trolox
TEREPHTHALIC assay	^●^OH	1549 ± 77 mmol equivalents DMSO	227 ± 14 mmol equivalents DMSO
ORAC assay	ROO^●^	156,472 ± 10,600 μmol equivalents Trolox	63,719 ± 3580 μmol equivalents Trolox

**Table 3:** Point zero of charge (PZC) and surface area (S_BET_) physicochemical properties for the 24 synthesized carbon supports. All assays were performed in triplicate n = 3. The same superscript letters for different materials indicate that the values are not statistically different.

Carbon Support	PZC	S_BET_ m^2^/g
DECs	9.8 ± 0.4 ^a^	646 ± 3 ^a^
DECsN	10.1 ± 0.3 ^a^	632 ± 1 ^b^
DECsP	2.5 ± 0.2 ^b^	317 ± 2 ^c^
DECsNP	4.4 ± 0.4 ^c^	549 ± 2 ^d^
DEBa	9.3 ± 0.4 ^a^	673 ± 3 ^e^
DEBaN	10.3 ± 0.3 ^a^	738 ± 2 ^f^
DEBaP	2.6 ± 0.2 ^b^	577 ± 1 ^g^
DEBaNP	3.6 ± 0.4 ^c^	540 ± 3 ^h^
IECs	10.0 ± 0.3 ^a^	652 ± 4 ^a^
IECsN	9.9 ± 0.2 ^a^	589 ± 1 ^j^
IECsP	2.4 ± 0.2 ^b^	455 ± 1 ^k^
IECsNP	2.6 ± 0.2 ^d^	348 ± 2 ^l^
IEBa	9.5 ± 0.3 ^a^	761 ± 4 ^m^
IEBaN	10.1 ± 0.2 ^a^	622 ± 3 ^n^
IEBaP	2.6 ± 0.2 ^b^	647 ± 4 ^a^
IEBaNP	2.9 ± 0.1 ^d^	614 ± 3 ^o^
PLCs	9.1 ± 0.4 ^a^	583 ± 2 ^g^
CsPLN	8.5 ± 0.2 ^e^	537 ± 3 ^h^
PLCsP	2.5 ± 0.2 ^b^	245 ± 5 ^p^
PLCsNP	2.9 ± 0.1 ^d^	301 ± 3 ^q^
PLBa	9.0 ± 0.4 ^a^	683 ± 2 ^r^
PLBaN	7.8 ± 0.1 ^f^	638 ± 2 ^s^
PLBaP	2.7 ± 0.2 ^b^	346 ± 5 ^l^
PLBaNP	2.9 ± 0.2 ^d^	566 ± 4 ^t^

## Data Availability

The original contributions presented in this study are included in this article. Further inquiries can be directed to the corresponding author/s.
